# Exploring human factors that affect patient safety in dentistry: a qualitative study of hospital-based practice

**DOI:** 10.1186/s12903-026-08921-3

**Published:** 2026-07-03

**Authors:** Minun Siddiqui, Minahil Aruj Younis, Ayesha Fahim, Ulfat Bashir, Farhana Ayub, Adnan Jehangir

**Affiliations:** 1 Islamic International Dental College, Riphah International University, Islamabad, Pakistan; 2 Tanner College of Dental Medicine, University of Pikeville, Pikeville, KY USA; 3 Biomedical Sciences Department, College of Medicine, King Faisal University, Al Ahsa, 31982 Saudi Arabia

**Keywords:** Human factors, Patient safety, Dentistry, SEIPS, Qualitative research, Low- and middle-income countries

## Abstract

**Introduction:**

This qualitative study explores how hospital-based dental professionals in Pakistan perceive and navigate human factors that influence patient safety, with particular attention to the structural and cultural contexts of low- and middle-income countries (LMICs) like Pakistan.

**Materials and methods:**

A qualitative exploratory design grounded in the SEIPS (Systems Engineering Initiative for Patient Safety) framework was employed. Purposive sampling was used to recruit participants with clinical experience in hospital-based dentistry. Interviews were audio-recorded, transcribed, and thematically analysed using Braun and Clarke’s framework, with deductive mapping to SEIPS domains. Member checking and coder triangulation supported analytical rigour.

**Results:**

Following interviews with 29 participants, three interrelated domains emerged: (1) Individual-level preconditions for safe practice, such as fatigue, emotional stress, and cultural sensitivity; (2) Team and process-level factors, including hierarchical barriers, communication gaps, and reliance on experienced support staff; and (3) Organisational and systemic conditions, including inadequate infrastructure, time pressure, and punitive supervisory cultures. These factors interact dynamically to affect clinical decision-making and procedural safety.

**Conclusion:**

Human factors shape patient safety in dental hospitals, particularly within LMIC contexts where structural limitations and cultural norms amplify existing risks. Addressing these challenges requires context-sensitive safety protocols, enhanced communication frameworks, ergonomic reforms, and supportive supervision. Findings underline the importance of integrating human factors education into dental curricula and governance to promote a culture of safety.

**Supplementary Information:**

The online version contains supplementary material available at https://doi.org/10.1186/s12903-026-08921-3.

## Key points

• Human factors such as fatigue, stress, and communication challenges can significantly influence patient safety in hospital-based dentistry.

• Improvements in infrastructure, communication protocols, and supervisory support can strengthen safety practices and reduce preventable harm

• Integration of human factors education into dental training and policy development is essential to promote a culture of safety in low-resource settings.

**Supplementary Information:**

The online version contains supplementary material available at https://doi.org/10.1186/s12903-026-08921-3.

## Introduction

In recent decades, patient safety has emerged as a key component of excellence and healthcare quality worldwide [1]. Patient safety is a multifaceted concept including prevention, identification, and mitigation of clinical errors and adverse events. The World Health Organization has identified preventable harm as the leading cause of morbidity and mortality in clinical settings, making patient safety a fundamental component of high-quality healthcare on a global scale [2]. Through the use of organised procedures, safety checklists, system redesigns, and a strong culture of reporting and learning from errors, the healthcare sector has made remarkable progress in enhancing patient safety over the last 20 years. Landmark initiatives such as the World Health Organization's Surgical Safety Checklist, the Institute for Healthcare Improvement’s “100,000 Lives Campaign,” and the implementation of root cause analysis in adverse event reporting have significantly reduced preventable harm in clinical settings [3]. Protocols like medication reconciliation, hand hygiene compliance, time-outs before procedures, and standardised communication tools like SBAR (Situation–Background–Assessment–Recommendation) have become routine in many hospitals across high-income countries, substantially lowering the rates of wrong-site surgeries, hospital-acquired infections, and diagnostic errors [4]. Despite these advances, dentistry remains lagging in the systematic integration of patient safety protocols. Literature shows that dental practices often lack standardised reporting systems for adverse events, and concepts like “near-misses” are under-recognised [5, 6].

Patient safety in dental care is influenced by a range of factors including complex clinical decision-making, time-sensitive procedures, and interprofessional coordination. Dentistry is distinguished from other medical specialties by specific procedural risks that necessitate tailored safety measures: dental sedation carries particular airway management challenges in the confined oral workspace; cross-infection control demands rigorous adherence to sterilisation protocols due to the aerosol-generating nature of high-speed handpieces and ultrasonic scalers; and the ergonomic demands of prolonged intraoral work impose musculoskeletal strains distinct from other surgical fields. These characteristics mean that universal safety frameworks designed for general medical environments often underserve dental settings.

In low- and middle-income countries (LMICs), these challenges are compounded by systemic vulnerabilities. While nations such as the UK, Australia, and Canada have incorporated incident reporting, continuing professional education, and human factors training into their healthcare systems through national safety agencies and accreditation bodies, LMICs face a markedly different reality [7, 8]. Countries including Bangladesh, Nigeria, and Pakistan contend with unreliable reporting systems, infrastructure constraints, and rigid clinical hierarchies that obstruct dialogue on patient safety [9, 10]. In Pakistan specifically, research demonstrates that the majority of hospitals lack standardised incident reporting methods and accredited safety training programs for dental professionals [11]. Similarly, a study in India found that institutional frameworks remained insufficient to support patient safety implementation in dental practices [12].

These gaps create a critical knowledge and implementation divide, leaving LMIC dental systems more vulnerable to preventable harm. It is in this context that exploring the perceptions and navigation strategies of hospital-based dental professionals becomes crucial, not only to understand the challenges, but to co-develop contextually viable solutions.

While these clinical, environmental, and systemic factors all matter, human factors stand out due to their pervasive impact on decision-making, communication, and the interface between people and systems. These factors, encompassing ergonomics, teamwork, decision-making, and organisational systems, have provided a powerful lens for understanding contributors to clinical errors [13]. However, there is a striking paucity of research examining how hospital-based dental professionals in LMICs perceive and navigate these human factors, and existing frameworks have been predominantly developed for resource-rich environments.

To conduct this investigation, we adopted the Systems Engineering Initiative for Patient Safety (SEIPS) model, which conceptualises healthcare delivery as the product of interactions among multiple system components, rather than individual actions alone [14]. The SEIPS model is particularly suited to the Pakistani context, where official policies often diverge from informal practices, making it well positioned to uncover concealed vulnerabilities and adaptive strategies that linear frameworks would miss. The framework's components and their application to this study are detailed in the Methods section.

Hence, this study aims to qualitatively explore how hospital-affiliated dental professionals in Pakistan identify, experience, and navigate key human factors that impact patient safety. This study advances knowledge in two ways: first, by generating context-specific evidence on human factors in LMIC dental practice, an area with minimal existing research; and second, by applying the SEIPS framework to hospital-based dentistry in an LMIC context, an application that has received limited attention in the existing literature.

## Materials and methods

### Study design

This study employed a qualitative exploratory approach grounded in the constructivist paradigm, which holds that knowledge is collaboratively developed through social interaction and practical experience. This paradigm was chosen because it foregrounds participants' subjective interpretations of their clinical environments, enabling exploration of how dental professionals construct meaning around patient safety within their specific cultural, institutional, and hierarchical contexts. The constructivist lens informed every stage of the research process: during data collection, open-ended interview questions were designed to elicit rich experiential narratives rather than standardised responses; during analysis, the research team engaged reflexively with the data, acknowledging that findings are co-constructed between researcher and participant; and during interpretation, emphasis was placed on understanding multiple perspectives rather than identifying a single objective truth. This approach was essential for investigating the nuanced interpersonal dynamics and systemic challenges that influence safety in LMIC dental hospitals.

### Theoretical framework

The Systems Engineering Initiative for Patient Safety (SEIPS) model served as the guiding theoretical framework. SEIPS views healthcare as a dynamic system shaped by interactions between five key components: (1) the person, including individual characteristics, skills, knowledge, and psychological state; (2) tasks, encompassing clinical demands, complexity, and cognitive load; (3) tools and technologies, including equipment, instruments, and information systems; (4) the physical environment, such as workspace design, ventilation, noise, and lighting; and (5) organisational conditions, including institutional culture, policies, staffing levels, and governance structures. By conceptualising patient safety as an emergent property of these interacting components, SEIPS moves beyond linear cause-and-effect models and enables identification of systemic vulnerabilities. In this study, SEIPS guided the development of interview questions to ensure coverage of all system components, directed the deductive phase of thematic analysis by mapping emergent codes to SEIPS domains, and provided an interpretive lens for understanding how individual, team, and organisational factors interact to shape safety outcomes. This structured alignment between framework and method ensured theoretical coherence throughout the research process.

### Study setting

Five dental teaching hospitals across multiple regions of Pakistan were selected as the research sites for this study. Data were collected from Islamic International Dental Hospital, Quetta Dental College, University of Lahore, Hi-Tech Dental College and Akhtar Saeed Dental College respectively. Only full-time employed staff were approached including consultants, PG trainees, junior dentists, and support staff.

### Sampling and participants

A purposive sampling technique was utilised to recruit participants with firsthand expertise in hospital-based dental care and involvement in patient safety decisions. Participants were eligible if they met the following inclusion criteria: (a) full-time employment at one of the five study sites; (b) a clinical role involving direct patient care, including consultants, postgraduate trainees, junior dentists, or dental support staff (dental technicians and dental assistants); and (c) a minimum of one year of clinical experience in hospital-based dentistry. Individuals were excluded if they were: (a) employed part-time or in a visiting capacity; (b) in administrative-only positions without clinical patient contact; or (c) employed at their current institution for less than one year, as they may have had insufficient exposure to the institutional safety culture. Participants were selected to reflect diversity in professional role, seniority, gender, and years of experience, enabling exploration of safety perceptions across system levels.

### Recruitment and consent

Potential participants were invited through departmental briefings and email communication. Each received an information sheet detailing the study’s purpose, voluntary nature, and underlying theoretical lens (SEIPS). Written informed consent was obtained for both participation and audio recording. Anonymity and confidentiality were guaranteed, and participants were reminded of their right to withdraw at any stage without consequence.

### Data collection

Data were collected through open-ended, one-on-one interviews conducted in private consultation rooms within the hospital to ensure comfort and confidentiality. The interview questions were developed by the researchers in accordance with AMEE guide no. 185 [15]. The questions were formulated to align with the SEIPS framework and existing literature on human factors in healthcare, ensuring both theoretical and contextual relevance. To establish content validity, the questions were reviewed by two independent experts in dental education and patient safety, and minor modifications were made based on their feedback. The finalised set of questions was then pilot-tested with two participants to assess clarity, comprehensibility, and contextual suitability before commencing full data collection (Supplementary file). Interviews were conducted over a four-week period, from 1 January to 1 February 2025. All sessions were audio-recorded with participant consent, and field notes were taken during and immediately after each interview. While most interviews were conducted in English, seven dental assistants who were less fluent in English preferred to respond in Urdu. For these interviews, a bilingual co-interviewer translated questions into Urdu and simultaneously interpreted participants' Urdu responses back into English for audio recording. The co-interviewer was trained in research ethics and well-versed in the study objectives to ensure accuracy and neutrality. Transcription was performed from the English audio recording. To verify accuracy, a second bilingual team member reviewed transcripts of translated interviews against the original audio recordings and field notes, and any discrepancies were discussed and resolved before analysis. Additional field notes captured contextual and cultural nuances not easily translatable. Data saturation was determined when no new codes emerged in two consecutive interviews.

### Transcription and member checking

Audio recordings were transcribed verbatim within one week of each interview. Transcripts were shared with participants for member checking, allowing them to verify the accuracy and intent of their responses. Minor edits were made based on feedback, enhancing the credibility and authenticity of the data.

### Data analysis

Data were analysed using Braun and Clarke’s six-phase thematic analysis framework, supported by NVivo 12 qualitative analysis software [16]. The analysis began with inductive coding, allowing themes to emerge organically from participants’ narratives without imposing pre-existing categories. This was followed by deductive mapping, where emergent themes were aligned with the SEIPS model domains and relevant constructs from human factors theory to ensure theoretical coherence. To enhance analytical rigour, a second coder independently analysed 25% of the transcripts, and any coding discrepancies were resolved through collaborative discussion. An audit trail was maintained throughout the process to systematically document analytical decisions, codebook development, and theme refinement, thereby supporting transparency and dependability in the analysis.

To enhance the trustworthiness of the study, we followed the four criteria established by Lincoln and Guba: credibility was ensured through member checking and coder triangulation; dependability was maintained by using a transparent and systematic coding protocol; confirmability was supported through the use of an audit trail; and transferability was achieved by providing thick descriptions of the research context and participant characteristics. The analysis thus followed a hybrid inductive–deductive approach: initial coding and theme development were grounded in participant narratives, after which themes were mapped to SEIPS work-system components. SEIPS was used as a sensitising and interpretive framework rather than as a rigid coding template, allowing the findings to remain empirically grounded while maintaining theoretical coherence.

### Researcher reflexivity

The research team maintained a reflexive stance throughout the study. The principal investigators (AF and UB) are clinical dental professionals and educators working within the Pakistani healthcare system. This insider positioning facilitated rapport with participants and contextual understanding of institutional dynamics, but also carried the risk of normalising systemic issues or interpreting findings through assumptions shaped by professional socialisation. To mitigate this, field notes and reflexive memos were maintained throughout data collection and analysis, capturing reflections on positionality, prior assumptions, and emergent interpretive decisions. Regular team debriefings were conducted to challenge each researcher's interpretations, and particular attention was paid to instances where the research team's own clinical experiences may have influenced coding or thematic development. The inclusion of co-investigators from outside the Pakistani dental system (FA and AJ) provided an additional external perspective during analysis and interpretation.

### Reporting standards

The reporting of this study follows the Consolidated Criteria for Reporting Qualitative Research (COREQ) guidelines, ensuring transparency and completeness in study design, data collection, analysis, and interpretation.

## Results

Because several findings crossed more than one SEIPS component, themes are presented according to their dominant qualitative meaning and then mapped to relevant SEIPS domains. Twenty-nine participants were interviewed, comprising consultants (*n* = 5), postgraduate trainees (*n* = 7), junior dentists (*n* = 6), and dental support staff (*n* = 11, including dental technicians and dental assistants). Participant ages ranged from 24 to 54 years, with 1 to 25 years of clinical experience. Interviews lasted between 30 and 60 min. Although qualitative thematic analysis studies typically include 12–20 participants, the larger sample of 29 was necessary to ensure adequate representation across five geographically diverse hospital sites and four distinct professional roles. Data saturation was confirmed at participant 27, with two additional interviews conducted to verify saturation. Participant demographics are presented in Table 1.

**Table 1:** Participant demographic information

Participant Group	n	Age Range (years)	Gender (Male/Female)	Roles/Positions Included
Consultants	5	45–54	3/2	Senior Consultants, Heads of Department
Post Graduate Trainees	7	32–36	5/2	Trainee Specialists
Junior Dentists	6	24–27	2/4	House Officers
Dental Support Staff	11	25–45	7/4	Dental Technicians (n = 4), Dental Assistants (n = 7)
Total	29	24–54	17/12	

Thematic analysis, guided by the SEIPS framework, revealed three interrelated domains influencing patient safety in hospital-based dental practice: (1) individual-level preconditions for safe practice, (2) team and process-level factors, and (3) organisational and system-level conditions (Table 2). Each theme and its subthemes were illustrated through participant narratives, derived inductively from the data and subsequently mapped deductively to SEIPS domains to ensure theoretical coherence.

**Table 2:** Themes, subthemes, and codes of human factors influencing patient safety in hospital-based dentistry

Theme (SEIPS Domain)	Subtheme	Codes (Examples)
Individual-Level Preconditions (Person Factors)	Fatigue and Ergonomic Strain	Fatigue; Sleep Deprivation; Hunger & Thirst; Ergonomic Strain
	Emotional & Cognitive Load	Stress & Anxiety; Emotional Resilience; Cognitive Load
	Cultural Sensitivity in Clinical Encounters	Gender norms; Patient comfort; Cultural inhibitions; Communication adaptation
Team and Process-Level Factors (Processes & Culture)	Competence and Anticipation by Support Staff	Anticipatory support; Error interception; Implicit teamwork; Dependence on individual competence
	Hierarchical Barriers to Speaking Up	Authority gradients; Fear of reprisal; Underreporting of near-misses; Power disparities
	Communication Clarity and Standardisation	Verbal miscommunication; Absence of handover protocols; Language barriers; Lack of SBAR
Organisational and System-Level Conditions (Environment & Structure)	Physical Infrastructure and Environmental Safety	Ventilation; Lighting; Cleanliness; Spatial adequacy; Sterilisation area design
	Time Pressure and Workload Overload	High Patient Load; Time Pressure; Skipped Breaks; Overbooking; Procedural shortcuts
	Supervisory Support and Feedback Culture	Punitive criticism; Lack of recognition; Resistance to change; Non-punitive reporting absent

### Theme 1: individual-level preconditions for safe practice (*SEIPS domain: Person factors)*

This theme reflects the individual conditions of dental professionals that shape their ability to provide safe care. Participants consistently emphasised that patient safety begins with the readiness and capacity of individual dental professionals, shaped by physical condition, cognitive focus, and emotional state.

#### Subtheme 1.1: fatigue and ergonomic strain

Clinicians described prolonged procedures and demanding schedules that resulted in physical discomfort, reduced attentiveness, and impaired precision. A consultant explained:

*“By the end of a long shift, my hands and shoulders are aching; I don’t feel I’m as sharp.”* (Consultant 2)

*“After back-to-back patients, you start losing focus… even simple steps feel harder to keep track of.”* (Junior Dentist 1)

*“I’ve seen doctors continue even when they look exhausted; you can tell their concentration is not the same.”* (Dental Technician 4)

These accounts suggest that fatigue operates as a latent condition for error, particularly during prolonged or repetitive clinical tasks. Importantly, participants did not frame fatigue as an individual failing, but as a consequence of systemic pressures such as staff shortages and overbooking that necessitate extended working hours. This reflects a clear interaction between organisational constraints and individual vulnerability within the SEIPS framework, where system-level inefficiencies directly compromise cognitive and physical performance.

#### Subtheme 1.2: emotional and cognitive load

Mental distraction, stemming from both personal stressors and workplace pressures, was repeatedly reported as undermining clinical decision-making:

*“If I’ve had an argument at home, I can’t concentrate in the clinic.”* (Junior Dentist 3)

*“Sometimes the pressure from seniors makes you second-guess your decisions, even when you know what to do.”* (Postgraduate Trainee 2)

*“If a mistake happens, the fear of being blamed stays in your mind during the next procedure.”* (Dental Assistant 6)

These narratives illustrate how emotional burden is not only internally generated but structurally reinforced through hierarchical expectations and punitive supervision. The persistence of fear and self-doubt reflects how organisational culture amplifies cognitive load, narrowing attention and impairing decision-making. Within the SEIPS model, this highlights how organisational and cultural conditions shape individual psychological states, creating downstream risks for patient safety.

#### Subtheme 1.3: cultural sensitivity and bias

Professionals shared their perspectives regarding the critical role of cross-cultural sensitivity in clinical encounters especially with regard to gender norms that influenced both patient comfort and quality of communication.

*“We have to be extra careful with female patients… always cautious to avoid making them uncomfortable.”* (Dental Technician 2)

*“Sometimes patients are hesitant to speak openly, especially with opposite-gender doctors, which makes diagnosis harder.”* (Junior Dentist 4)

*“We often have to adjust how we communicate depending on the family or cultural background of the patient.”* (Postgraduate Trainee 5)

These perspectives indicate that cultural dynamics extend beyond interpersonal sensitivity to directly influence information exchange and clinical clarity. Communication adjustments were often necessary but inconsistent, suggesting the absence of structured cultural competence strategies. This reveals how sociocultural context functions as an embedded system factor within SEIPS, shaping both patient interaction and clinical decision-making.

### Theme 2: team and process-level factors (SEIPS domains: Processes and organisational culture)

This theme highlights the interpersonal and communication component of patient safety. Team interactions were an important determinant of safe practice exhibiting both strengths (facilitating collaboration) and weaknesses (suppression of hierarchy and disrupted communication).

#### Subtheme 2.1: competence and anticipation by support staff

Participants described experienced dental support staff as an important informal safety buffer within busy clinical workflows. Their anticipatory actions, preparing instruments, recognising clinicians' needs, and noticing potential errors helped maintain procedural flow and reduce confusion.

*“My assistant knows what I need before I ask, this prevents delays and confusion.”* (Consultant 1)

*“A good assistant can catch small mistakes before they happen, especially when things get hectic.”* (Junior Dentist 2)

*“We prepare instruments in advance because we know delays can frustrate the doctor and affect the procedure.”* (Dental Assistant 7)

These accounts reinforce the role of experienced support staff as an informal safety mechanism within clinical workflows. However, reliance on individual anticipation rather than standardised protocols suggests variability in safety performance. From a SEIPS perspective, this highlights a process-level adaptation compensating for gaps in formal system design.

#### Subtheme 2.2: hierarchical barriers to speaking up

Organisational hierarchies were perceived by participants across role groups as a hindrance to voicing their concerns and challenging the appropriate authorities. A junior dentist reflected:

*“You don’t want to be the one always questioning the boss.”* (Junior Dentist 2)

*“As a trainee, you notice issues but you hesitate because you don’t want to seem disrespectful.”* (Postgraduate Trainee 3)

*“Even when we see something wrong, we usually stay quiet unless asked directly.”* (Dental Assistant 3)

These findings demonstrate how hierarchical gradients suppress upward communication across multiple staff levels, not just among junior clinicians. The normalisation of silence reflects a deeply embedded cultural norm that limits error interception. Within the SEIPS framework, this represents a breakdown in team processes driven by organisational culture, increasing the likelihood of unmitigated risks.

#### Subtheme 2.3: communication clarity

Ambiguities in verbal instructions occasionally led to near-miss events:

*“I said to pass the syringe and they passed the wrong one; it nearly led to an error.”* (Consultant 3)

*“Sometimes instructions are rushed, and you assume what the doctor meant instead of confirming.”* (Dental Technician 5)

*“In busy clinics, we don’t always repeat or clarify instructions, which can lead to confusion.”* (Postgraduate Trainee 6)

These accounts highlight how time constraints and informal communication practices contribute to ambiguity in clinical interactions. The reliance on assumption rather than verification reflects the absence of structured communication protocols. This underscores a process-level vulnerability within SEIPS, where inefficient communication systems increase the risk of near-miss events.

### Theme 3: organisational and system-level conditions (SEIPS domains: Environment, tools, organisational structures)

The third theme highlights the broader organisational and systemic environment in which dental care is delivered. At the systems level, participants described structural and environmental challenges that shaped the broader context for safety, often beyond their direct control.

#### Subtheme 3.1: physical infrastructure and environmental safety

Participants highlighted deficiencies in infrastructure, ventilation, cleanliness, and lighting, that compromised both safety and professional performance:

*“Dust blows in when the window’s open; it’s not hygienic but we have no choice.”* (Dental Technician 1)

*“Sometimes the equipment isn’t functioning properly, but we still have to continue with the procedure.”* (Junior Dentist 6)

*“The workspace gets crowded, and it becomes difficult to maintain proper sterility.”* (Dental Assistant 8)

These narratives illustrate how infrastructural deficiencies directly constrain safe clinical practice. Participants frequently adapted to suboptimal environments, indicating normalisation of risk. Within SEIPS, these environmental limitations represent upstream system failures that shape frontline behaviours and safety outcomes.

#### Subtheme 3.2: time pressure and workload overload

High patient volumes created a sense of urgency that led to procedural shortcuts and missed breaks. A junior dentist shared:

*“I skip breaks just to keep up; it’s no surprise mistakes happen.”* (Junior Dentist 5)

*“We are expected to see so many patients in limited time, so you end up rushing procedures.”* (Postgraduate Trainee 4)

*“There’s constant pressure to keep the queue moving, even if it means cutting corners.”* (Dental Assistant 9)

These accounts suggest that time pressure is structurally embedded within institutional practices, particularly overbooking and inadequate workforce planning. Rather than isolated individual decisions, rushed care emerges as a predictable response to systemic demand–capacity mismatches. This demonstrates how organisational policies propagate downstream risks at the task and individual levels within the SEIPS framework.

#### Subtheme 3.3: supervisory support and feedback culture

Participants described a culture where feedback was often punitive rather than constructive, reducing morale and discouraging safety-related dialogue:

*“I had worked non-stop for three days and my boss just criticised me; I felt demoralised.”* (Junior Dentist 4)

*“Feedback usually comes only when something goes wrong, not when things are done correctly.”* (Postgraduate Trainee 1)

*“We feel discouraged to report issues because it might lead to blame instead of support.”* (Dental Assistant 10)

These findings reflect a predominantly punitive feedback culture that undermines psychological safety and open communication. The emphasis on blame rather than learning discourages reporting and limits opportunities for system improvement. Within SEIPS, this highlights how organisational culture shapes team behaviours and suppresses mechanisms essential for safe practice.

## Discussion

This study qualitatively explored how hospital-based dental professionals in Pakistan perceive and navigate human factors that influence patient safety. Using the SEIPS framework, three interrelated domains were identified: individual-level preconditions for safe practice, team and process-level factors, and organisational and system-level conditions. Collectively, these findings suggest that patient safety in dental hospitals is shaped not only by technical competence, but also by the interplay of personal well-being, team culture, and structural conditions. Importantly, the study provides novel insights from an LMIC context where human factors in dentistry remain under-researched, extending global patient safety discourse beyond the hospital wards and operating theatres where it has been traditionally centred.

These three domains interact dynamically within the SEIPS model: individual-level factors such as fatigue and cognitive load are both caused by and contribute to organisational-level conditions such as understaffing and workload overload; team-level communication breakdowns reflect both individual hesitancy shaped by cultural norms and organisational hierarchies that suppress open dialogue; and infrastructure deficits at the system level directly constrain individual performance and team coordination. This reciprocal interaction explains why isolated interventions targeting only one system level are insufficient, effective safety improvement requires addressing all levels simultaneously.

Our findings align with international evidence demonstrating the risks posed by fatigue, ergonomic strain, and emotional stress in healthcare. In dentistry, researchers have shown how musculoskeletal discomfort and prolonged standing impair procedural accuracy, a concern echoed by participants in our study [17]. Emotional and cognitive load, exacerbated by hierarchical pressure, mirrors reports from medical contexts where burnout and mental distraction increase error risk [18]. However, this study highlights the compounded impact of these factors in Pakistani dental hospitals, where staffing shortages and lack of structured breaks intensify physiological strain.

Interpersonal dynamics, especially hierarchy and communication challenges, mirrored findings from high-income countries, where authority gradients often discourage junior staff from speaking up and hinder open dialogue [19]. However, in Pakistani context these issues were even more pronounced, shaped by cultural norms that discourage questioning senior clinicians and result in near-misses going unreported [20]. At the same time, participants highlighted the crucial role of competent auxiliary staff, whose ability to anticipate clinicians’ needs often acted as a safety buffer [21]. Similar patterns have been reported in Saudi Arabia and the UK, where experienced assistants help prevent delays and errors [22, 23]. What stands out in Pakistan, however, is that such support frequently substitutes for the absence of formal safety protocols, rather than serving as a complement to them.

At the organisational level, participants described deficits in infrastructure, infection control, and supervisory culture. Similar patterns have been reported in India, Nigeria, and Bangladesh, where LMIC dental institutions face overcrowding, weak governance, and underinvestment in safety infrastructure [24–26]. What distinguishes this study is the identification of a punitive supervisory culture: participants described demoralisation following criticism and the absence of constructive feedback. While punitive approaches have been widely critiqued in global patient safety literature, this study suggests they remain entrenched in LMIC dentistry, limiting opportunities for collective learning. Another unique dimension was the role of gender and cultural sensitivity, which influenced both patient interactions and intra-team communication. Although gender bias in dental education has been described in Pakistan, its influence on safety practices in clinical settings has been largely overlooked [27].

These organisational findings must be understood within the broader health system context. In Pakistan, dental teaching hospitals operate within a healthcare system characterised by chronic underfunding of primary and secondary dental care, absence of a national dental patient safety policy, fragmented regulatory oversight across provincial health departments, and workforce shortages that create structural incentives for overbooking and procedural shortcuts [28–30]. Unlike countries with mature patient safety infrastructures, such as the United Kingdom's National Health Service which mandates incident reporting through a national framework, Pakistan lacks a centralised mechanism for reporting, analysing, or learning from dental adverse events [31]. This regulatory vacuum means that even well-intentioned individual and institutional efforts operate without systemic support, explaining why safety practices in our study were largely dependent on informal workarounds rather than embedded protocols.

Taken together, these findings suggest that patient safety in LMIC dental hospitals is highly contingent on individual resilience and informal teamwork rather than robust organisational systems. This warrants critical reflection: while such adaptations represent genuine strengths, over-reliance on them reflects underlying organisational failure. Three key insights emerge. First, cultural norms around gender, hierarchy, and communication shape safety behaviours in ways under-recognised by existing frameworks. Second, structural factors, understaffing, high patient volumes, and lack of mandated rest periods, drive individual stress and fatigue at the system level. Third, LMIC safety practices frequently depend on informal workarounds that underscore both the system's resilience and its fragility.

These findings highlight the urgency of incorporating study of human factors into dental education, hospital administration, and dental practice in LMICs. Achieving this requires several targeted steps. To begin with, structured communication protocols such as SBAR can be utilised to ensure consistent team handover practices and to minimise uncertainty. Subsequently, to ensure clinician performance, various techniques must be employed to reduce ergonomic strain and fatigue through optimised clinic design, adequate rests and occupational health initiatives. Furthermore, creating supportive supervisory culture is a prerequisite for promoting learning and accountability by replacing harsh criticism with constructive dialogue and recognition of safe practices. To better align practices with WHO guidelines patient safety measures such as covering emergency planning, infection control, and sterilisation must be formally adopted. Ultimately, funding workforce strengthening and training initiatives would fortify their role as proactive safety providers, ensuring that this contribution is formally recognised and supported through training and instruction. The WHO Global Patient Safety Action Plan (2021–2030), which advocates for systemic changes to improve patient safety capability throughout LMIC health systems, is in line with these measures taken together [2].

This study's strengths include its theory-driven design using the SEIPS framework, the inclusion of diverse professional roles across five dental teaching hospital sites in Pakistan, and the use of member checking and coder triangulation to enhance credibility. However, several limitations should be acknowledged. First, although data were collected from five institutions, these were all teaching hospitals, and findings may not fully represent private practice or non-teaching settings. Second, the use of real-time translation for interviews with support staff may have introduced subtle interpretive bias, despite cross-checking with bilingual team members and field notes. Third, social desirability bias may have led participants to underreport negative experiences or personal errors, particularly in a context where hierarchical norms discourage open disclosure. Fourth, the representation of professional roles was unequal: dental support staff constituted the largest group (*n* = 11) while consultants were fewest (*n* = 5), which may weight findings toward certain perspectives. Fifth, as a qualitative study, findings are not intended to be statistically generalisable; however, the thick descriptions provided and the diversity of sites enhance analytic transferability to similar LMIC dental contexts.

Further research is needed to evaluate interventions targeting identified human factors. Comparative studies across multiple LMIC settings could assess whether gender norms, hierarchical silencing, and infrastructural deficits are regionally specific or broadly generalisable. Quantitative studies could measure the prevalence of fatigue, burnout, and miscommunication events in dental hospitals, while intervention trials could evaluate the effectiveness of structured communication protocols, supervisory training, and ergonomic redesigns in improving safety outcomes. Incorporating patient perspectives on safety, particularly regarding cultural and gender dynamics, is also essential to capture the full spectrum of safety risks in dental care.

## Conclusion

This study suggests that patient safety in LMIC dental hospitals is shaped by the interaction of individual, team, and organisational factors rather than by individual effort alone. The findings have implications for practice, including the incorporation of human factors training into dental education, implementation of incident reporting systems and constructive supervisory frameworks at the institutional level, and workforce planning that addresses staffing adequacy and scheduled rest periods. These findings contribute to the limited evidence base on human factors in LMIC dental settings and may inform safety improvements in dental teaching hospitals facing similar resource constraints.

## Supplementary Information

Supplementary Material 1.

## Data Availability

The qualitative datasets generated and analysed during the current study are not publicly available due to the sensitive and potentially identifiable nature of interview transcripts, which could compromise participant confidentiality despite anonymisation. The datasets are available from the corresponding author on reasonable request, subject to appropriate ethical approval.

## Abbreviations

SEIPS: Systems Engineering Initiative for Patient Safety
LMIC: Low- and middle-income country
SBAR: Situation–Background–Assessment–Recommendation
WHO: World Health Organization
COREQ: Consolidated Criteria for Reporting Qualitative Research
