# Supplementary material for: Exploring human factors that affect patient safety in dentistry: a qualitative study of hospital-based practice
Source: BMC Oral Health. 2026 Jul 3;26:1799. doi: 10.1186/s12903-026-08921-3 (PMC13599177; doi:10.1186/s12903-026-08921-3)
Supplement: Supplementary file 1 [file 12903_2026_8921_MOESM1_ESM.pdf]

Title:
Exploring the Human Factors Involved in Dentists' Performance in a Hospital Setting

Interview guide

Thank you for taking the time to participate in this study titled *"Exploring Human Factors That Affect Patient Safety in Dentistry: A Qualitative Study of Hospital-Based Practice."* The purpose of this research is to understand how individual, team, and organizational factors influence patient safety in hospital-based dental settings. Your insights will help identify challenges and practical strategies to improve safety and quality of care in dentistry.

Participation in this interview is entirely voluntary. You may choose not to answer any question or withdraw from the interview at any time without any consequence. By agreeing to take part and continuing with the interview, you are providing your consent to participate in this study.

Your responses will be treated with strict confidentiality. No names or identifying information will be recorded or reported, and all data will be analyzed and presented anonymously. The information you share will be used solely for academic and research purposes.

The interview will last approximately 30–60 minutes. Please feel free to speak openly about your experiences and perspectives, there are no right or wrong answers.

Section 1. Opening questions:

- Can you describe your current role and main clinical responsibilities in your department?
- How long have you been working in a hospital-based dental setting?
- How do you personally define *patient safety* in your day-to-day practice?

Section 2. Individual-Level Preconditions for Safe Practice

- What personal or individual factors do you think most influence your ability to provide safe dental care?
- How do physical factors such as fatigue, ergonomics, or workload affect your clinical focus or precision?
- Can you describe any situations where emotional stress or mental distraction influenced your performance?
- How does your personal life, including family or social responsibilities, affect your ability to maintain concentration or performance in the clinic?

- Do gender, age, or cultural expectations influence how you interact with patients or colleagues? If so, how?

Section 3. Team and Process-Level Factors (SEIPS Domain: Processes & Organizational Culture)

- How would you describe teamwork and communication within your dental unit?
- Can you give an example of how communication (good or poor) affected patient safety in your setting?
- How do hierarchies or authority gradients influence whether team members feel comfortable speaking up about safety concerns?
- In what ways do experienced support staff, such as dental assistants or nurses, contribute to or hinder safe practice?
- What training or informal practices help ensure coordination and understanding within the team?

Section 4. Organizational and System-Level Conditions (SEIPS Domain: Environment & Structure)

- How does the physical environment of your hospital (e.g., lighting, ventilation, layout) affect safety and workflow?
- How do factors such as staffing levels, patient volume, or scheduling influence your ability to provide safe care?
- What kinds of equipment or technological limitations do you experience that affect patient safety?
- How would you describe the supervisory or feedback culture in your institution?
- What organizational supports, or lack thereof, help or hinder you in maintaining safe dental practices?

Section 5. Closing Reflection

- What, in your opinion, are the most important changes that could improve patient safety in hospital-based dental practice?
- Is there anything else you would like to add about your experience regarding patient safety or human factors?
